# Tumor-Associated Macrophages as Incessant Builders and Destroyers of the Cancer Stroma

**DOI:** 10.3390/cancers3043740

**Published:** 2011-09-28

**Authors:** Manuela Liguori, Graziella Solinas, Giovanni Germano, Alberto Mantovani, Paola Allavena

**Affiliations:** 1 Department of Immunology and Inflammation Istituto Clinico Humanitas, Via Manzoni 113, Rozzano-Milano 20089, Italy; E-Mails: manuela.liguori@humanitasresearch.it (M.L.); graziella.solinas@ucsf.edu (S.G.); giovanni.germano@humanitasresearch.it (G.G.); alberto.mantovani@humanitasresearch.it (M.A.); 2 Department of Translational Medicine, University of Milano, Milano 20089, Italy

**Keywords:** Tumor-Associated Macrophages (TAM), extra-cellular matrix (ECM), stroma, proteases, MMPs, cancer, inflammation

## Abstract

Tumor-Associated Macrophages (TAM) are key components of the reactive stroma of tumors. In most, although not all cancers, their presence is associated with poor patient prognosis. In addition to releasing cytokines and growth factors for tumor and endothelial cells, a distinguished feature of TAM is their high-rate degradation of the extra-cellular matrix. This incessant stroma remodelling favours the release of matrix-bound growth factors and promotes tumor cell motility and invasion. In addition, TAM produce matrix proteins, some of which are typical of the neoplastic tissues. The gene expression profile of TAM isolated from human tumors reveals a matrix-related signature with the up-regulation of genes coding for different matrix proteins, as well as several proteolytic enzymes. Among ECM components are: osteopontin, osteoactivin, collagens and fibronectin, including also a truncated isoform of fibronectin termed migration stimulation factor. In addition to serve as structural proteins, these matrix components have key functions in the regulation of the vessel network, in the inductionof tumor cell motility and degradation of cellular debris. Among proteolytic enzymes are: matrix metalloproteases, cathepsins, lysosomal and ADAM proteases, and the urokinase-type plasminogen activator. The degrading activity of TAM, coupled to the production of bio-active ECM proteins, co-operate to the build-up and maintenance of an inflammatory micro-environment which eventually promotes tumor progression.

## 1. Introduction

Mononuclear phagocytes are essential cells for wound healing and tumors can be described as wounds that never heal [1]. Macrophages are numerous in the stroma of experimental and human tumors and mediate important biological functions that profoundly affect tumor cell behaviour. By secreting a number of diverse chemoattractants, primary tumors recruit blood circulating monocytes; here they differentiate to Tumor-Associated Macrophages (TAM) primarily because of the presence of M-CSF produced by neoplastic cells. Conditioned by the local milieu (rich in IL-10, TGFβ and prostaglandins), they acquire immune-suppressive and pro-tumoral effector properties. TAM are key players in cancer-related inflammation; with their continual deposition and degradation of the extracellular matrix (ECM) TAM actively contribute to the build-up the typical reactive micro-environment of tumors.

The interstitial matrix is an intricate and highly dynamic network of fibres composed of glycosaminoglycan (GAG)-containing glycoproteins. Different types of fibrous collagen together with fibronectin, hyaluronan and proteoglycans confer mechanical strength, elasticity and a precise spatial organization to tissues. In addition, the extracellular basement membrane, a specialized form of sheetlike ECM mainly composed by collagen IV and laminins, is very important to sustain the epithelial cell layer and maintain orientation of apicobasal polarity [2]. Besides structural support, the interstitial matrix and basement membranes are important to integrate complex signalling and to regulate cellular movement, proliferation and differentiation [3,4]. Furthermore, the ECM contains a wide range of growth factors that are bound in an inactive form to matricellular proteins, but can be rapidly released and activated in case of need, for example during tissue repair [5-7].

Neoplastic cells modify their stroma and vasculature through production and secretion of different growth factors and cytokines. The locally changed host microenvironment, in turn, regulates the proliferation and invasive behaviours of tumor cells. The tumoral ECM presents several different features compared to normal tissue ECM, not only for the presence of aberrantly expressed or modified structural proteins but also, and most importantly, because of the incessant remodelling operated by several proteolytic enzymes. ECM degradation has several important consequences: altered stiffness and composition of the ECM; fragmentation of basement membranes, which facilitates the motility and invasive ability of tumor cells; deregulated organization of the vessel network. All these processes, initially guided by tumor cells and gradually involving the contribution of host cells, lead to the construction of a reactive stroma where the cross-talk and signalling between the diverse cell types and ECM proteins is outside the normal control. Here we will review the role of tumor macrophages in neoplastic tissues, in particular their important contribution to the continuous remodelling of the tumor stroma.

## 2. Significance of Macrophages in Tumors

Macrophages are versatile cells that are capable of displaying different functional activities, some of which are antagonistic; for instance they can be immuno-stimulatory or immuno-suppressive, and either promote or restrain inflammation [8,9]. This functional plasticity is regulated by local cues to which the macrophages respond. For instance during bacterial infections macrophages first orchestrate the acute inflammatory response and eliminate the invading pathogens; at later time points they transform into scavengers of tissue debris, and finally trigger the proliferative phase of healing by releasing a variety of growth factors and cytokines which recruit and activate fibroblasts and new vessels [10-15].

Macrophage heterogeneity has been simplified in the macrophage polarization concept where the two extreme phenotypes, the M1 and M2 macrophages, have distinct features. M1 or classically–activated macrophages are stimulated by bacterial products and Th1 cytokines (e.g., IFNγ); they are potent effectors that cope bacterial infections and may have cytotoxic activity towards transformed cells [16,17]. M2 or alternatively activated macrophages differentiate in micro-environments rich in Th2 cytokines (e.g., IL-4, IL-13); they have high scavenging activity, produce several growth factors that activate the process of tissue repair and suppress adaptive immune responses [18-20].

**Figure 1.**
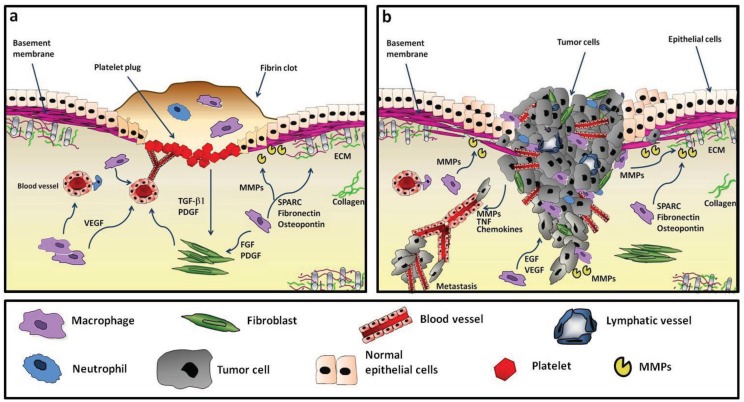
Comparison between wound healing and the reactive tumor microenvironment. Wound repair after tissue injury (**a**) is characterized by platelet aggregation, migration of leukocytes (neutrophils and macrophages) to the site of injury and by production of growth factors and cytokines involved in neo-angiogenesis and cell proliferation (e.g., PDGF, VEGF, FGF, TGFβ), and in ECM remodeling proteases (e.g., MMPs, SPARC, Fibronectin), thereby promoting wound healing and resolution. In the tumor microenvironment; (**b**) similar factors are produced by tumor-associated macrophages, fibroblasts and cancer cells, but neither with temporal control nor in a regulated manner. The continuous expression of stimulating growth factors and of proteolytic enzymes leads to a reactive milieu and enhanced angiogenesis that support tumor cell survival, proliferation and invasion of surrounding tissues.

In established tumors, TAM resemble M2-like macrophages [21-23]. While M2-related activities are of extreme importance during wound healing to return to the homeostatic state, in the context of a growing tumor they may favour disease progression [16,17,21,24-27]. Indeed, neoplastic tissues show similarities to sites of tissue repair (Figure 1).

TAM are poorly cytotoxic against neoplastic cells and, instead, have been shown to influence fundamental aspects of tumor biology. Among the well documented pro-tumor functions of TAM is the production of many growth factors for tumor cells and for the nascent blood and lymphatic vessels, which are essential for the neo-angiogenesis switch and tumor proliferation. These include for instance epidermal, fibroblast and vascular growth factors (EGF, FGF, VEGF) [25,28-30]. TAM are also a major source of proteolytic enzymes that degrade the extra-cellular matrix thus favouring the invasion of neoplastic cells [25,31]. They contribute to the evasion of tumors from immune control by producing immune-suppressive cytokines such as IL-10 and TGF-beta [25,32].

In line with the above evidence, high density of TAM has been significantly associated with poor prognosis in the majority of tumors [17,20,25,33]. Indeed, markers of macrophages or their products are present in the stroma-associated gene signature predicting clinical outcomes (see below).

Some studies in human colorectal cancer, however, indicated that macrophages may have anti-tumor activity [34-36]. TAM localization appears of primary importance: the number of peritumoral macrophages, but not of those within the cancer stroma, was associated with improved disease-free survival. Peritumoral macrophages had higher expression of costimulatory molecules (CD80 and CD86) and were able to induce apoptosis in cancer cells by a Fas ligand-dependent mechanism [37,38]. It may be possible that by being less exposed to tumor-derived immune-suppressive cytokines outer macrophages are able to differentiate into cytotoxic effectors.

## 3. Features of the ECM of Tumors

In normal tissues resident stromal cells (fibroblasts, leukocytes, endothelial cells) are typically quiescent. In tumors, the contrast is macroscopically evident, as observed by pathologists over 100 years ago. The tumor stroma is highly inhomogeneous with more abundant ECM, activated fibroblasts, irregular vessels and numerous inflammatory leukocytes [39-41].

In the normal stroma each cell type displays surface receptors appropriate to its environment. ECM proteins function cooperatively to modulate the interaction between different cellular components, basement membranes and interstitial matrix proteins. These processes keep under strict control various cellular processes such as growth, death, adhesion, migration, gene expression and differentiation, and are of relevance either to maintain homeostasis and to cope tissue repair in case of injury [4,42].

The tumor stroma is characterized by a remarkable subversion of the tissue architecture, especially in poorly differentiated carcinoma, and by a different composition of some ECM components. Ultrastructural and immunohistochemical analyses revealed the up-regulation of several proteins such as tenascin, decorin, byglican, α-smooth muscle actin, osteopontin, fibulin-1, fibronectin, and the appearance of spliced protein isoforms that are not normally expressed [4,43-45]. While the vascular network in normal tissues is characterized by regular dichotomous branching, the tumor vasculature is disorganized, with numerous capillary branches, blind buds or dilated vessels, and a decreased number of pericytes. Indeed, neoplastic tissues appear to be in a constant state of tissue damage [43,46-48].

Probably the most remarkable characteristic of tumoral stroma is the high level of proteolytic degradation [49-51]. This phenomenon has several consequences: first, it alters stroma stiffness and removes the physical barriers between cells, facilitating the invasion of migrating cells (neoplastic and endothelial cells); second, cleaved ECM proteins may reveal cryptic sites and generate abnormal signalling; third, ECM-stored growth factors are released in active form and directly stimulate tumor cell survival, proliferation, motility and the neo-angiogenic switch.

### Stromal Signature and Prognosis

Altered expression of genes related to the ECM has been studied in association with patient clinical outcome in a number of human tumors [52-54]. The expression of VEGF and MMP7 predicted the risk of poor prognosis in hepatocellular carcinoma [55]. In stomach cancer, the transition from pre-invasive to invasive lesions was characterized by the up-regulation of stromal and inflammatory genes. The signature associated with adverse clinical outcome included: TGF-related genes (*thrombospondin 1*); metalloproteases (*MMP1*); junction-mediating and regulatory protein (*JMY*); markers of stromal activation: fibroblast activation protein alpha (*Fap-α*) [56]. A study in diffuse large-B-cell lymphoma revealed two interesting signatures that were more highly expressed in the non-malignant fraction and predicted survival in patients. The genes defining the first stromal signature encoded ECM components such as fibronectin, Secreted Protein Acidic and Rich in Cysteine (SPARC), various collagen and laminin isoforms, modifiers of collagen synthesis and several matrix proteases. The second stromal signature encoded endothelial cell-related genes: the von Willebrand factor, CD31, CXCL12 and VEGFR2 [57].

Macrophage-related gene signatures have been identified in human tumors such as ovarian and breast cancer, soft tissue sarcoma and follicular B lymphoma [57-60]; in classic Hodgkin's lymphoma, tumors with increased number of CD68+ TAM were significantly associated with primary treatment failure and shortened progression-free survival [61].

Further, TAM and related myeloid cells with immune suppressive functions (MDSC) [62-64] and the pro-angiogenic Tie-2 monocytes [65] have been implicated in the failure to anti-tumor therapies [66,67] via mechanisms that included the secretion of the myeloid cell–dependent angiogenic factor Bv8 [68].

## 4. Matrix Degradation and Remodelling

Even in normal tissues the ECM is not a static structure and microscopic changes are determined by a careful balance between matrix synthesis, secretion, modification and enzymatic degradation. Such dynamic remodeling is amplified, in a deregulated manner, in tumor tissues. Matricellular proteins are degraded by specific proteases which can be grouped in large families and include matrix metalloproteases (MMPs), cathepsins, hyaluronidases, ADAM proteases, but also heparanase, elastase, urokinase-type plasminogen activator (uPA), plasmin and others [69,70].

Tumors have high turnover of ECM proteins and protease activity. Although neoplastic cells and fibroblasts are able to produce proteolytic enzymes, macrophages are considered the major cell type expressing protease activity in tumor tissues [50,71,72]. Immunohistochemical and enzymatic analyses in different tumors have shown that increased expression of proteases or changes in cell localization are important prognostic factors which correlate with tumor progression [51,73] Proteolysis of ECM proteins disrupts integrin-mediated anchorage and focal adhesion kinase (FAK) and is pivotal for cancer cell invasion into the adjacent space [31,74-76].

TAM and their released factors (e.g., IL-1 and TNF) have long been known to augment tumor metastasis [77,78]. In addition they are an important source of proteolytic enzymes, especially MMPs and uPA [49,50,79]. Of note, TAM produce several chemokines which beyond regulating cell motility–are able to activate MMPs [80,81]. The role of TAM in cancer cell invasion has been visualized in experimental tumors *in vivo* by multiphoton microscopy; by using fluorescently labelled cells Wyckoff and colleagues showed that tumor cell intravasation occurs next to perivascular macrophages in mammary tumors [82,83]. Further, it has been recently shown that the cathepsin protease activity of IL-4-stimulated TAM promotes tumor invasion [84]. IL-4 is produced by tumor-infiltrating CD4 T cells and there is mounting evidence of its relevance in the polarization of macrophages with pro-tumor functions [85,86].

Cleavage of matrix molecules also reveals available binding sites that were previously masked to cell surface receptors, and fragments with new functional effects. For instance, MMP-2 degradation of collagen unveils integrin-binding sites that rescue melanoma cells from apoptosis [47] while the trimeric NC1 domain of collagen XVIII induces angiogenesis [87]. Cryptic epitopes of fibronectin trigger angiogenesis and tumor growth [88,89].

Over the last decade there has been recognition that proteins of the ECM can modulate multiple functions of innate immune cells. A cryptic peptide of laminin-10, a prominent component of basement membranes, is chemotactic for neutrophils and macrophages and induces the up-regulation of TNF, chemokines and MMP-9 [90]. Particular attention has been given to proteolytic ECM fragments and the activation of Toll-like receptors: versican activates TLR2 and TLR6 on TAM and stimulates the production of IL-6 and TNF, two prototypic cytokines of cancer-related inflammation [91]. Hyaluronan fragments induce the expression of inflammatory genes in immune cells through activation of TLR4 and TLR2 as well as the CD44 receptor [92]. Thus ECM glycoproteins and glycosaminoglycans can directly stimulate inflammatory cells and contribute to fuel inflammation at tumor sites.

**Figure 2.**
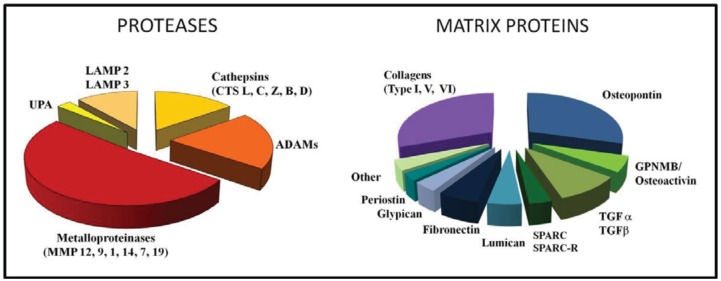
Gene expression profile of human tumor-associated macrophages. The data refer to the expression of genes coding for proteolytic enzymes or ECM proteins. Results are mean values from 7 different TAM preparations. Each depicted slice is proportional to the expression level of each gene (Affymetrix).

We recently performed an Affymetrix gene profiling of TAM isolated from human ovarian carcinoma and found that among the most up-regulated genes were several genes coding for ECM proteins or related to its remodelling (Figure 2). Among proteolytic enzymes, the most expressed were MMPs (12, 9, 1 and 14), Cathepsins (L,C,Z and B), uPA, lysosomal enzymes and ADAM proteases (Figure 2).

The matrix is a valuable repository of growth factors: members of the EGF and FGF families, TGF-beta and related members, as well as PDGF and VEGF, bind to the various components of ECM and are stored, in an inactive form, until released and activated by matrix proteases. In the tumor context, increased proteolytic activity releases active growth factors which stimulate tumor and stromal cells [47,50,93,94].

For example, MMPs, plasmin and heparanase degrade the angiogenic factor FGF-beta [95]. MMP-3 has been shown to cleave the matrix molecule decorin, thereby delivering active TGF-beta [96] and MMP13 appears to be essential for the release of VEGF from the ECM in squamous cell carcinoma [97]. Of note, during ECM proteolysis fragments with angiostatic activity can also be generated. Thus the ultimate biological response really depends on the balance between pro-and anti-angiogenic factors.

Degraded matrix proteins need to be eliminated. A major function of macrophages, dictated by their name, is the phagocytosis of apoptotic cells and cellular debris, and their final disposal in specialized lysosomal compartments. As mentioned above, TAM expresses high levels of lysosomal-enriched cathepsins, which facilitate the elimination of ingested proteins.

### Osteoactivin

One of the most up-regulated genes in our TAM profiling, as well as in macrophages co-cultured with tumor cells [98], codes for the protein osteoactivin, whose function is not completely characterized but appears to play a role in tissue repair after injury. Osteoactivin, also called glycoprotein non-metastatic melanoma protein B (GPNMB) or haematopoietic growth factor inducible neurokinin-1 (HGFIN), was originally identified in osteoblasts/osteoclasts as a critical mediator of differentiation, bone remodelling and turnover [99], and in myeloid DC where it negatively regulated T cell activation [100]. Studies in tumors showed that it is over-expressed in various malignant tumors such as breast cancer, melanoma, glioma, and is involved in the promotion of angiogenesis and tumor invasiveness [101,102].

A recent report, however, uncovered a novel activity and demonstrated that osteoactivin is essential after renal tissue injury for the disposal of cellular debris and appropriate healing [103]. This protein is localized on the cell membrane and contains an Arg-Gly-Asp integrin-binding domain, important for cell adhesion; upon cleaveage by ADAM10 it is shed into the surrounding milieu [104]. Its role in tissue remodelling and repair was already suggested by the finding that in osteoactivin-transgenic mice, it showed a cytoprotective effect on the fibrosis induced by skeletal muscle denervation, via a mechanism related to the up-regulation of MMP3 and MMP9 [105]. In ischemic renal injury, osteoactivin is up-regulated in damaged epithelial cells, but is much higher in infiltrating macrophages. Gpnmb mutant mice had decreased repair of the kidney and macrophages showed many more undigested apoptotic cellular debris compared to wild-type mice. In phagosomes, Osteoactivin co-localizes with the autophagy protein LC3, and later in lysosomes for final degradation [103]. These findings show that osteoactivin is a phagocytic protein produced by macrophages, essential for the disposal of injured tissues.

## 5. Matrix Deposition by TAM and Their Relationship with Fibroblasts

Fibroblasts are master regulators of matrix deposition in the stroma and are influenced by stimuli coming from both inflammatory cells (macrophages) and neoplastic cells. Several growth factors produced by TAM are able to activate fibroblasts: EGF, FGF, PDGF and, above all, TGF-beta. In turn, activated fibroblasts (or myofibroblasts, as they start producing a-smooth muscle actin) release growth factors for epithelial cells (IGF, EGF, HGF), chemokines for macrophages (CCL2, CXCL12) and activated MMPs [28,29,106]. Thus, tumor-associated fibroblasts are key cells of the reactive tumor micro-environment. In addition, also TAM are very active producers of matricellular proteins. TAM contribute to matrix architecture by producing for instance osteopontin, fibronectin, proteoglycans, SPARC and different collagen types [14,15,107].

### 5.1. Osteopontin

Several ECM-related genes were expressed in our gene-profiling from human TAM (Figure 2). The top expressed gene was osteopontin; this protein is a component of the ECM being a secreted protein and is produced also by stromal and tumor cells [108-110]. Osteopontin has multiple functions in tumors being involved in protease activation and ECM remodelling, cell adhesion and migration, angiogenesis, as well as in inflammation and immunity [107,111-114]. Serum levels of osteopontin are elevated in cancer patients and usually correlate with tumor progression, raising the issue of its clinical use as a potential biomarker [108,115,116].

The involvement of this ECM protein has been demonstrated in several aspects of malignancy. A correlation between osteopontin up-regulation and malignant invasion was suspected because this protein controls cell motility and invasion through the engagement of CD44 receptors and integrins [110,117]. It was also involved in the accelerated proliferation of indolent tumors, via the pro-tumoral role of bone marrow-derived leukocytes, that are recruited by Osteopontin and activated to produce inflammatory cytokines [118]. A pro-migratory effect has been demonstrated also on endothelial cells. Osteopontin-mediated matrix degradation, achieved by the activation of MMP9 and uPA, indirectly promotes the neo-angiogenic switch [111,112,119,120]. Therefore, cell-ECM adhesion, inhibition of apoptosis and induction of migration are crucial functions mediated by osteopontin that eventually promote tumor cell survival and dissemination.

### 5.2. Migration Stimulation Factor

Among the classical ECM proteins that we found up-regulated in TAM and in tumor-conditioned macrophages was fibronectin [98]. Spliced isoforms of fibronectin, also called oncofoetal isoforms ED-A and ED-B, are known to be increased in tumors and during embryogenesis [121-124]. A number of studies have reported their role in promoting endothelial cell migration and tumor angiogenesis [43,123,124]. We found that tumor macrophages expressed a third oncofetal FN isoform, known as Migration Stimulation Factor (MSF) [98]. MSF is a truncated isoform of fibronectin identical to the 70-kDa N terminus but with a unique 10 aa sequence. MSF was cloned in 2003 by Schor and colleagues who demonstrated its potent motogenic activity on fibroblasts [125]. Of interest, Macrophage-secreted MSF potently stimulated the in vitro migration of tumor cells, as well as monocytes [98]. This truncated fibronectin isoform is not an exclusive product of TAM, being produced also by neoplastic cells and vascular endothelial cells [125,126]. Of note, MSF is expressed in vitro only by M2 macrophages and is down-regulated in M1 cells. Thus, MSF may represent a good candidate marker of the M2-type polarization of TAM.

### 5.3. SPARC

SPARC is another matricellular glycoprotein highly expressed at sites of tissue remodelling. SPARC regulates the interactions between cells and their microenvironment, mediating matrix deposition and turnover, cell adhesion and signaling by extracellular factors. In neoplastic tissues, SPARC is expressed in the stroma and in malignant cells of some types, and affects tumor development, metastasis, angiogenesis and inflammation. SPARC-induced changes can suppress or promote progression of different cancers depending on the tissue and cell type. In some cancers, such as melanomas and gliomas, SPARC is associated with a highly aggressive tumor phenotype, while in others, mainly ovarian cancer, neuroblastoma and colorectal cancer, SPARC may function as a tumor suppressor [127-129].

A major function of SPARC is its involvement in collagen deposition, as demonstrated in tumors transplanted in Sparc-/- mice: growing tumors showed reduced collagen fibers and decorin deposition. In addition SPARC binds to other components of the ECM and of the basement membranes, such as entactin/nidogen and thrombospondin 1, and therefore contributes to the organization of the interstitial matrix. Probably because of this altered matrix, pancreatic tumors grown orthotopically in Sparc-/-mice were more metastatic than tumors grown in wild-type mice [130,131].

The relationship of SPARC and the immune system has also been studied in tumors. In tumor-bearing Sparc-/- mice there was a reduced macrophage recruitment suggesting that SPARC may have chemotactic activity on macrophages [132,133]. Of interest, TAM and M2-polarized macrophages express the SPARC receptor, Stabilin-1, a scavenger receptor that targets SPARC for lysosomal degradation [134]. Thus a reciprocal feedback control is envisaged: SPARC recruits macrophages which, in turn, express stabilin-1 that clear SPARC from the environment.

Another SPARC-related loop, interconnecting tumor biology and immunity, was demonstrated by Sangaletti *et al.* They analyzed the respective roles of host- and tumor-derived SPARC in wild-type and SPARC-/- mice using bone marrow chimeras. It turned out that SPARC produced by infiltrating leukocytes, rather than by the tumor, was instrumental in appropriate deposition of collagen IV in peritumoral stroma from mammary carcinoma, whereas reciprocal chimeras (SPARC-/- bone marrow cells in wild type mice) developed tumors with less defined lobular structures [135]. The data underlie the importance of SPARC (produced by host leukocytes) in the assembly and organization of tumor stroma. Further, the same group showed that SPARC produced by TAM enhances cancer cell migration and spontaneous metastasis, via a mechanism that involved avb5 integrin [136].

## 6. Targeting of TAM in Tumors

As summarized above, TAM functional activities importantly contribute to the construction of the reactive tumor micro-environment and are, therefore, amenable targets of biological therapies. Macrophage depletion in experimental settings has been successful in decreasing tumor growth and metastatic spread [11,137,138]; furthermore their depletion may contribute to a better response to conventional chemotherapy and anti-angiogenic therapy [62,63,65-67]. Several approaches have been followed to target TAM in tumors such as inhibition of their recruitment at tumor sites or the use of cytotoxic drugs, for example, biphosponates.

A number of studies have shown that the bisphosphonate clodronate-encapsulated in liposomes is an efficient reagent for the depletion of macrophages. Clodronate-depletion of TAM in tumor-bearing mice resulted in reduced angiogenesis and decreased tumor growth and metastasis [139]. Moreover, the combination of clodronate with sorafenib significantly increased the efficacy of sorafenib alone in a xenograft model of hepatocellular carcinoma. In clinical practice, bisphosphonates are employed to treat osteoporosis; current applications in cancer treatment include their use to treat skeletal metastases in multiple myeloma, prostate and breast cancer. Treatment with zoledronic acid was associated with a significant reduction of skeletal-related events and, possibly, direct apoptotic effects in tumor cells [140-142].

Another approach is to inhibit the recruitment of circulating monocytes in tumor tissues. Among the many chemokines expressed in the tumor micro-environment, CCL2 (or monocyte chemotactic protein-1) occupies a prominent role and has been selected for therapeutic purposes. Pre-clinical studies have shown that anti-CCL2 antibodies or antagonists to its receptor CCR2, given in combination with chemotherapy, were able to induce tumor regression and yielded to improved survival in prostate mouse cancer models [143-145].

A third and more recent approach is to ‘re-educate’ TAM to exert anti-tumor responses protective for the host, ideally by using factors able to switch the M2-phenotype of TAM into that of M1-macrophages with potential anti-tumor activity. This was achieved in experimental mouse tumors by injecting the TLR9 agonist CpG- oligodeoxynucleotide (CpG-ODN), coupled with anti-IL-10 receptor [146] or the chemokine CCL16 [147]. CpG-ODN synergized also with an agonist anti-CD40 mAb to revert TAM displaying anti-tumor activity [148]. A remarkable anti-tumor effect of re-directed macrophages has been recently reported in human pancreatic cancer with the use of agonist anti-CD40 mAb [149]. A recent report showed that the plasma protein histidine-rich glycoprotein (HRG), known for its inhibitory effects on angiogenesis [150,151] is able to skew TAM polarization into M1-like phenotype by down-regulation of placental growth factor (PlGF), a member of the VEGF family. In mice, HRG promoted anti-tumor immune responses and normalization of the vessel network [152].

The intense protease activity present within tumors has been the object, over several years, of pharmaceutical research, looking for specific MMPs inhibitors [49,50]. The first generation of developed compounds were competitive inhibitors (e.g., batimastat), and later were derivatives of tetracycline, which inhibited MMP gene transcription and enzymatic activity. Of note, also the biphosponates inhibit MMP activity. When tested in clinical trials several years ago, these compounds gave overall disappointing results. The field, however, is still active and considering the use of monoclonal antibodies specific for membrane-bound MMPs, such as MMP14 [51,153].

Few years ago, by studying a new anti-tumor agent of marine origin, trabectedin, we unexpectedly observed that this compound was highly cytotoxic to monocytes and macrophages, with a remarkable selectivity, as neutrophils and lymphocytes were not killed [154]. Trabectedin has now been registered in 2007 in Europe for the treatment of soft tissue sarcoma, and in 2009 for ovarian cancer [155-157]. Trabectedin also affects gene transcription with a peculiar selectivity; some inflammatory cytokines and chemokines are reduced by the drug, such as IL-6, CCL2, CXCL8, VEGF, while TNF is not inhibited [158]. Another affected gene is collagen [159]. We therefore tested whether other ECM-related genes produced by macrophages were reduced by the drug. In *in vitro* experiments with monocytes/macrophages we found that low non-cytotoxic concentrations of trabectedin significantly decreased the expression of both the full length fibronectin and MSF. Also osteopontin and MMP2 were inhibited, while osteoactivin was not (Liguori, unpublished data). These results indicate that trabectedin may reduce the high turnover of the tumor stroma. This effect of “normalization” of the micro-environment, combined with its cytotoxic effect on macrophages and tumor cells, makes trabectedin an interesting compound in oncology.

## 7. Conclusions

In the last decades the concept that the ECM is simply a supporting structure for the preservation of tissue architecture has dramatically changed [160]. Indeed, ECM components provide signals affecting cell adhesion, migration, proliferation and differentiation. In particular, degraded/proteolytic fragments of ECM molecules or their aberrant expression, as occurs during neoplastic transformation, can sustain the activation of inflammatory cells. TAM are key players of the cancer-related inflammation present at tumor sites. In addition to releasing cytokines and growth factors, a distinguished feature of TAM is their high rate of remodelling of the tumor stroma, in which they vigorously participate by expressing proteolytic enzymes and ECM proteins, some of which are specific to the neoplastic tissues. Such a reactive micro-environment eventually supports tumor cell proliferation and the full-blown development of neo-angiogenesis. There is increasing evidence that successful anti-cancer therapies are not only dependent on the cancer phenotype but also on the normalization of the tumor stroma. In this view, depletion of the unfaithful TAM, or their “re-education”, may contribute to the success of conventional anti-tumor therapies.
